# Comparison of 25-hydroxyvitamin D and Calcium Levels between Polycystic Ovarian Syndrome and Normal Women 

**DOI:** 10.22074/ijfs.2015.4201

**Published:** 2015-04-21

**Authors:** Ashraf Moini, Nooshin Shirzad, Marzieh Ahmadzadeh, Reihaneh Hosseini, Ladan Hosseini, Shahideh Jahanian Sadatmahalleh

**Affiliations:** 1 Department of Gynecology and Obstetrics, Arash Women’s Hospital, Tehran University of Medical Sciences, Tehran, Iran; 2 Department of Endocrinology and Female Infertility at Reproductive Biomedicine Research Center, Royan Institute for Reproductive Biomedicine, ACECR, Tehran, Iran; 3 Vali-e-Asr Reproductive Health Research Center, Tehran University of Medical Sciences, Tehran, Iran; 4 Endocrinology and Metabolism Research Center, Endocrinology and Metabolism Clinical Sciences Institute, Endocrinology and Metabolism Research Institute, Tehran University of Medical Sciences, Tehran, Iran; 5 Department of Reproductive Health and Midwifery, Faculty of Medical Sciences, Tarbiat Modares University, Tehran, Iran

**Keywords:** Polycystic Ovary Syndrome, 25-Hydroxyvitamin D, Calcium

## Abstract

**Background:**

Given the relationship of vitamin D deficiency with insulin resistance syndrome as the component of polycystic ovary syndrome (PCOS), the main aim of this study was to compare serum level of 25hydroxyvitamin D [25(OH)D] between PCOS patients and normal individuals.

**Materials and Methods:**

A cross sectional study was conducted to compare 25(OH)D level between117 normal and 125 untreated PCOS cases at our clinic in Arash Hospital, Tehran, Iran, during 2011-2012. The obtained levels of 25(OH)D were classified as follows: lower than 25 nmol/ml as severe deficiency, between 25-49.9 nmol/ml as deficiency, 50-74.9 nmol/ml as insufficiency, and above 75 nmol/ml asnormal. In addition, endocrine and metabolic variables were evaluated.

**Results:**

Among PCOS patients, our findings shows 3(2.4%) normal, 7(5.6%) with insufficiency, 33(26.4%) with deficiency and 82(65.6%) with severe deficiency, whereas in normal participants, 5(4.3%) normal, 4(3.4%) with insufficiency, 28(23.9%) with deficiency and 80(68.4%) with severe deficiency. Comparison of 25(OH)D level between two main groups showed no significant differences (p= 0.65). Also, the calcium and 25(OH)D levels had no significant differences in patients with overweight (p=0.22) and insulin resistance (p=0.64). But we also found a relationship between 25(OH)D level and metabolic syndrome (p=0.01). Furthermore, there was a correlation between 25(OH)D and body mass index (BMI) in control group (p=0.01), while the C-reactive protein (CRP) level was predominantly higher in PCOS group (p<0.001).

**Conclusion:**

Although the difference of 25(OH)D level between PCOS and healthy women is not significant, the high prevalence of 25(OH)D deficiency is a real alarm for public health care system and may influence our results.

## Introduction

Polycystic ovarian syndrome ( PCOS ) is known as one of the most common endocrine disordersand affects 5-10% of women that is characterized by hyperandrogenism and chronic anovulation. PCOS as a multi-dimensional syndrome influences various systems. Infertility, irregular menses, acanthosis nigricans, insulin resistance, and hirsutism are known as some of symptoms of PCOS (1). Also, it has some long term consequences such as hypertension coronary artery diseases and type II diabetes. Therefore, PCOS seems a real dilemma for gynecologists and endocrinologists to reveal its basic phathophysiology and offer a reasonable treatment. 

There are many evidence showing the relationship between serum level of 25hydroxyvitamin D [25( OH )D] and PCOS. Furthermore, the strong association between PCOS and insulin resistance indicates that insulin directly influences ovarian function (2), while impaired glucose tolerance and insulin secretion have been shown to be associated with vitamin D deficiency (3). Additionally, recent data have suggested that both calcium and vitamin D supplements may improve insulin sensitivity in PCOS women (4). 

There are suggestions that calcium has important role in activation and maturation of oocyte in animals (5); therefore, abnormalities in calcium metabolism may play an important role in pathogenesis of PCOS. 

In this study, we tried to investigate the correlation between vitamin D levels and PCOS in our population in order to make a decision about screening programs or supplement therapy in PCOS patients. Also, we aimed to find a correlation between body mass index ( BMI ), hyperandrogenism and metabolic syndrome with serum level of 25( OH )D in PCOS patients. 

## Materials and Methods

This cross sectional study and was performed on 242 women, 125 patients with PCOS and 117 healthy individuals, during 2011-2012. All women in 16-44 age group ( reproductive age ) were recruited from our clinic in Arash Hospital, Tehran, Iran, consecutively. PCOS was diagnosed based on the presence of two of following Rotterdam criteria: oligo and/or anovulation, clinical and/or biochemical signs of hyperandrogenism, and polycystic ovaries in ultrasound, meaning presence of 12 or more follicles measuring 2-9 mm in diameter in each ovary and/or ovarian volume more than 10 cm ^3^ The main problems of PCOS patients who attended our clinic were abnormal uterine bleeding and infertility.

A healthy woman was defined as woman in reproductive age with regular cycles. They came to our clinic for annual check-up, or their partners had male fertility problems. Women with congenital adrenal hyperplasia, hyperprolactinemia, hyperparathyroidism, and androgen secretory tumors were excluded using specific laboratory tests to verify the concentrations of 17 OH progesterone, dehydroepiandestronsulfate ( DHEAS ), and prolactin. Furthermore, women who used medications suspicious to affect carbohydrate metabolism or calcium/vitamin D concentrations during 6 months prior to the study, who had a history of chronic disease or endocrinophaties, and who had a history of smoking or drug abuse were excluded. All participants were living in Tehran, Iran, and they had no history of calcium or vitamin D supplementation. 

A morning blood sample was taken after 12 hours fasting during the follicular phase ( 3-5 days of spontaneous or progesterone-induced menstrual cycle ). The levels of calcium, vitamin D, insulin, high density lipoproteincholesterol ( HDL-C ), fasting blood sugar ( FBS ), triglyceride ( TG ), thyroid stimulating hormone ( TSH ), prolactin, testosterone, dehydroepiandrosterone sulfate ( DHEAS ), and C-reactive protein ( CRP ) were measured. All samples were obtained during fall and winter seasons. 

The PCOS patients were divided into two subgroups as follows: metabolic syndrome group ( n=39 ) and non-metabolic syndrome group ( n=86 ). The metabolic syndrome was defined by the National Cholesterol Education Program ( NCEP ) and the adult treatment panel III ( ATP III ) after observing three of the four following criteria: systolic blood pressure >130 mmHg and diastolic blood pressure >85 mmHg, TG level >150 mg/dl, HDL <50 mg/dl, fasting glucose >100 mg/dl, and waist circumference >88 cm. Also, we used the homeostatic model assessment of insulin resistance ( HOMA-IR ) to evaluate insulin resistance based on the following formula: fasting plasma glucose ( mmol/L )×fasting plasma insulin ( µU/L ) divided by 22.5. 

Next 25( OH )D was measured using enzymelinked immunosorbent assay ( ELISA, IDS, Boldon, UK ) with normal range of 75-100 nmol/ ml with 5.4 coefficient of variation ( CV ). Furthermore, insulin ( 2.0-25.0 μIU/ml ), prolactin ( 2.8-29.2 μg/ml ), TSH ( 0.1-4 mIU/ml ), total testosterone ( 0.14-0.9 ng/ml ), and DHEAS ( up to 5.8 µg/ml ) concentrations were measured using ELISA ( Monobind, USA ). Fasting glucose ( ≤100 mg/dl ), TG ( ≤150 mg/dl ), HDL ( >40 mg/ dl ), calcium ( 8.3-10.5 mg/dl ) and CRP ( ≤8 mg/ml ) concentrations were determined using photometry ( Parsazmoon, Iran ). Hyperandrogenism was defined as the clinical presence of hirsutism ( Ferriman-Gallway score ≥8 ), acne or alopecia and/or elevated androgen levels, meaning as testosterone level above 0.9 ng/ml and/or DHEAS level above 5.8 µg/ml. The measurement of 25( OH )D concentration was done using serum assay and at least 1 cc of patient’s serum was stored in freezer ( -40˚C ) for maximum 30 days. 

The approval was obtained from the Ethic Committee of Endocrinology and Metabolism Research Institute, Tehran University of Medical Science, Tehran, Iran, and an informed consent was obtained from all participants. 

### Statistical analysis

We used Kolmogorov-Smirnov test ( K-S test ) for evaluating data distribution. To analyze differences between groups, Student’s t test was used for normally distributed samples and nonparametric MannWhitney U-test was appliedfor abnormally distributed samples. Relationship between variables were evaluated using Pearson’s correlation coefficient. All analyses were performed by SPSS version 16 ( SPSS Inc., Chicago, IL, USA ). A value of p<0.05 was considered as statistically significant. 

## Results

Totally, all data were collected from 125 PCOS patients and 117 healthy women. The mean age was 27.85 in PCOS group and 30.82 in normal subjects. Also, the mean weight in PCOS and healthy women were 83.08 and 82.97, respectively. Table 1 shows the means of various factors in two main groups. 

**Table 1:** Comparison of different biological and biochemical values between two main groups


	PCOS Mean±SD	Non-PCOS Mean±SD	P value

**Age (Y)**	28.2± 8.4	30.82± 7.12	0.02
**BMI (kg/m^2^)**	25.92± 4.71	24.41± 3.88	0.01
**FBS (mg/dl)**	90.33± 16	90.99± 9.47	0.61
**TG (mg/dl)**	119.41±66.73	111.12±49.72	0.77
**HDL-C (mg/dl)**	48.24± 13.7	47.7±9.49	0.82
**Ca (mg/dl)**	9.34± 0.92	9.43±0.69	0.88
**CRP (mg/ml)**	1.77± 1.97	1.50±2.08	<0.001
**Testosterone (ng/ml)**	0.82± 0.37	0.82±0.41	0.46
**TSH (mIU/ml)**	2.55± 2.02	2.30±1.87	0.163
**DHEAS (μg/ml)**	1.59± 0.80	1.47±0.76	0.18
**Insulin (µIU/ml)**	16.66± 17.3	15.34± 11.19	0.96
**Prolactin (ng/ml)**	15.96± 10.2	16.55± 9.45	0.41
**25-OH vitamin D (nmol/ml)**	8.92± 6.43	9.29±7.35	0.41


BMI; Body mass index, FBS; Fasting blood sugar, TG; Triglyceride, HDL; High density lipoprotein, CRP; C-reactive protein, TSH; Thyroid stimulating hormone and DHEAS; Dehydroepiandrosterone sulfate.

We stratified level of 25( OH )D as follows: lower than 25 nmol/ml as severe deficiency, between 25-49.9 nmol/ml as deficiency, 50-74.9 nmol/ml as insufficiency, and above 75 nmol/ ml as normal (6). Among PCOS patients, our findings shows that 3 ( 2.4% ) normal, 7 ( 5.6% ) with insufficiency, 33 ( 26.4% ) with deficiency and 82 ( 65.6% ) with severe deficiency, whereas in normal participants, 5 ( 4.3% ) normal, 4 ( 3.4% ) within sufficiency, 28 ( 23.9% ) with deficiency and 80 ( 68.4% ) with severe deficiency. 

Totally, 162 women ( 66.9% ) had severe deficiency, 61women ( 25% ) had deficiency and 11 women ( 4.5% ) had insufficiency, whereas only 8 women ( 3.3% ) were normal. Comparison between two main groups showed no statistically differences. The mean of calcium was 9.34 mg/ dl in PCOS subjects and 9.43 mg/dl in normal group. None of them implied statistical significant differences. Table 2 shows the relationships between 25( OH )D and other parameters in PCOS and non-PCOS groups. 

**Table 2:** The correlation of vitamin D and other biochemical levels between two main groups


	25-hydroxyvitamin D					
	PCOS group			Non-PCOS group		
	R	P	OR	R	P	OR

**BMI**	-0.63	0.50	-0.02	0.10	0.25	-0.13
**FBS**	-0.56	0.55	-0.06	-0.05	0.52	0.23
**TG**	-0.08	0.36	0.24	0.18	0.04	-0.14
**HDL-C**	-0.10	0.25	-0.08	-0.16	0.06	0.00
**Ca**	-0.01	0.90	0.08	0.10	0.24	0.19
**CRP**	-0.06	0.52	-0.08	-0.03	0.69	-0.05
**Testosterone**	-0.05	0.55	-0.04	0.00	0.95	0.19
**TSH**	0.06	0.49	0.09	0.03	0.66	-0.07
**DHEAS**	0.00	0.99	0.22	0.17	0.92	0.01
**Insulin**	-0.11	0.23	-0.11	0.00	0.94	-0.07
**Prolactin**	0.06	0.51	-0.17	-0.14	0.10	-0.31


BMI; Body mass index, FBS; Fasting blood sugar, TG; Triglyceride, HDL; High density lipoprotein, CRP; C-reactive protein, TSH; Thyroid stimulating hormone, DHEAS; Dehydroepiandrosterone sulfate, P; P value, R; Pearson correlation coefficient and OR; Odd ratio.

**Fig.1:**
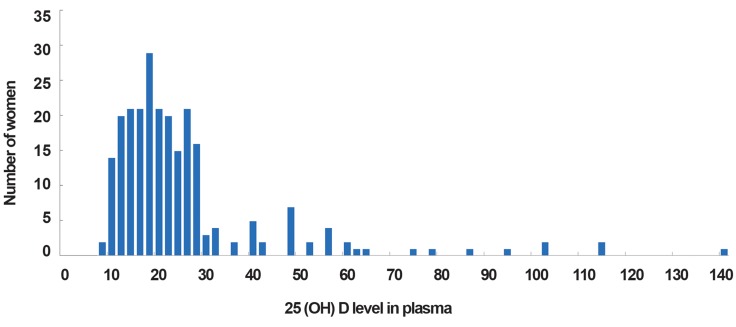
Distribution of 25( OH )D level in women.

We chose consecutive patients in our clinic, and the age and BMI values were different between two main groups in final analysis. We used logistic regression to control confounding factors and in this analysis, PCOS was not correlated with 25( OH )D level ( OR: 1, 95% CI=0.971, p=0.65, Fig.1 ). 

Furthermore, in PCOS group, 39 women belonged to metabolic syndrome group, while86 women belonged to non-metabolic syndrome group. The mean of 25(OH)D and calcium concentrations were 28.15 nmol/ml and 9.27 mg/dl in the metabolic syndrome group in comparison with 22.57 nmol/ml and 9.39 mg/dl in the non-metabolic syndrome group, indicating no significant difference between two sub-groups (p_vitD_=0.18, p_Ca_=0.35). Also, in control group, the levels of Ca and 25(OH)D showed no statistically difference (p_vitD_=0.59, p_Ca_=0.17). But in total population of our study, there was a relationship between metabolic syndrome and the level of 25(OH)D (p_vitD_=0.01, p_Ca_=0.35). According to BMI, 59 out of 125 PCOS patients (47.2%) were lean, 39 patients (31.2%) were overweight, and 27 women were obese (21.6%). The mean of calcium and vitamin D concentrations were 25.57 nmol/ml and 9.33 mg/dl in normal weight women, 24.34 nmol/ml and 9.34 mg/dl in overweight women, and 15.29 nmol/ml and 9.39 mg/dl in obese women with presenting no significant differences between these sub-groups(p_vitD_=0.87, p_Ca_=0.52). Although, the level is lower in obese women. In control group, the level of 25(OH)D is significantly lower in obese women (p_vitD_=0.01, p_Ca_=0.47). In total population, the level of 25(OH)D showed no significant correlation with BMI (p=0.22).

We had 62 patients with insulin resistance in PCOS (HOMA-IR >2.5) group and the mean of their 25(OH)D and calcium concentrations were 25.08 ng/ml and 9.32 mg/dl,respectively. Besides, 63 women were non-insulin resistant with the mean of 23.95 ng/ml for 25(OH)D level and 9.38 mg/ml for calcium level, showing there were no significant differences between these two subgroups (p_vitD_=0.37, p_Ca_=0.74).

A total of 30 patients had hyperandrogenism and the mean of their calcium and 25( OH )D concentrations did not differ significantly with the related values of non-hyperandrogenism sub-group. 

C-reactive protein, as an inflammatory factor, was measured and showed no correlation with 25-hydroxyvitamin D levels; however, it was significantly higher in PCOS patients. 

## Discussion

In overall, the most prominent point in our study was the high percentage of moderate to severe 25( OH )D deficiency in over than 70% of our population. It seems that this high prevalence influenced other aspects of our study. This proportion is lower in other studies, like 26.7% in PCOS patients in Germany (7), 2.9% in Austria (8), 27% in Caucasian infertile women (9), and 64.9% for female population in Iran (10). 

Animal researches have demonstrated the role of calcium in oocyte activation and maturation (5) and hypothesized that disturbances in calcium homeostasis may mediate the pathogenesis of PCOS. In our study, there was a high incidence of 25( OH )D deficiency in both PCOS and normal groups. Although the PCOS group had lower levels of calcium and 25( OH )D, the difference was not considerable. Also, Yildizhan et al. (11) evaluated 100 patients with PCOS and found no correlation between 25( OH )D level and PCOS, but they suggested a negative correlation between BMI and 25( OH )D levels. In our study, this negative correlation was found only in control group. It may be due to confounding effects of other factors such as higher rate of insulin resistance and metabolic syndrome in PCOS group. 

In another study from Hahn et al. (7) who evaluated 120 PCOS patients, there was a significant negative correlation between 25( OH )D levels and BMI, but there was no difference in calcium level. 

In addition, Asheim et al. (12) studied consecutively 76 PCOS women and 30 healthy ones and found lower levels of 25( OH )D in morbidly obese women. 

25( OH )D deficiency has diverse effects in human bodies. There is evidence that demonstrate a correlation between 25( OH )D and insulin resistance. Although the mechanisms underlying these associations are not fully understood (13,14), vitamin D has some effects on beta-cell function and may have a beneficial effect on insulin action by stimulating the expression of insulin receptors (8). In a study by Hahn et al. (7), they showed that 25( OH )D was associated with higher BMI values and body fat. In severely obese patients, Manco et al. (15) has illustrated that the fat mass is the best predictor for serum level of 25( OH )D. However, the mechanisms mediating these finding remain unclear. 

Despite these facts, in this study, the level of 25( OH )D did not differ significantly between two sub-groups ( obese and non-obese ), in total. It may be due to high percentage of severe vitamin D deficiency in our subjects that influenced our outcomes. 

Furthermore, our result imply a negative correlation between metabolic syndrome and 25( OH )D level which is in agreement with mentioned mechanisms of 25( OH )D. According to study of Wehr et al. (8) which included 205 PCOS women, they found a strong association between low serum levels of vitamin D and the metabolic syndrome. Also, we saw a positive correlation between TG and 25( OH )D levels in PCOS group ( p=0.002 ). It may be due to the common nutritional sources, but this result has not been reported in other articles and needs more precise studies. 

Another study in Iran also shows that 64.9% of women in Tehran have mild to moderate 25( OH ) D deficiency (11). Also, several studies from the Middle East have implied high prevalence of vitamin D deficiency in this area (16,19). Possible explanation for high proportion of vitamin D deficiency is different level of sun exposure as a result of urban life style or different levels of calcium intake. Low-calcium, high-phytate diets, pregnancy, and winter-related reduced sunlight exposure have been reported as probable causes (16). 

Furthermore, many studies have reported negative correlation between 25( OH )D level and HOMA-IR (8,11,20). 25( OH )D is believed to have some roles in insulin release, expression of insulin receptors, and suppression of cytokines that are possible mediators for insulin resistance (21). But in this study, although the HOMA-IR ( as insulin resistance ) was higher in severe deficient group, the difference was not statistically significant. It may be due to the small sample size or high percentage of deficiency in all groups. 

Whereas obesity and insulin resistance aggravate hyperandrogenism (7), there are no significant differences in testosterone and DHEAS levels between PCOS and non-PCOS women in our study. Therefore, the correlation between 25( OH ) D and testosterone levels in our PCOS group is not reliable. Perhaps this result can be due to the fact that we didnot measure the sex hormone binding globulin ( SHBG ) and free androgen index ( FAI ) in this study. Some data revealed a significant correlation between levels of 25( OH )D with SHBG and FAI (7,20). The total androgen level is not significantly higher in PCOS groups. Some PCOS studies in Iran have reported 20-40% hyperandrogenism in native population with PCOS (22,23) and some other studies from Middle East have found lower androgen levels than expected (24). But we need to reevaluate the androgen level of our PCOS patients in another study with different methods of measurement. 

Besides, in our study, PCOS group showed higher levels of CRP level and BMI that have been known as risk factors for cardiovascular disease ( CVD ), leading to elevated risk of CVD and/or stroke in this population. Women with PCOS are characterized by a prothrombotic state, as reflected by increased plasminogen activator inhibitor1( PAI-1 ) activity and fibrinogen concentration. The inflammatory markers such as CRP together with low heart rate explain high fibrinogen levels in women with PCOS (25). In other studies, this elevation has been also reported (26,27). In a study by Li et al. (20), they reported that 25( OH )D concentration was negatively correlated with CRP. However, in this study, we did not find this correlation ( e.g. higher level of CRP in sever vitamin D deficiency group ). 

There are increasing evidence indicating the role of vitamin D deficiency as a risk factor for multiple sclerosis (28), type 1 diabetes (29), CVD (30), and several malignant tumors (31). So, this pandemic of 25( OH )D deficiency is an alarm for public health care system and implies an emergent need to interfere. Consequently, we should try to change people’s life style and to design practical plans for food fortification and screening programs. Our study had some limitations such as relatively small sample size and no information available with respect to dietary calcium intake. 

It seems that there is an emergent need for supplement therapy and screening programs among our women in reproductive age-PCOS and -nonPCOS groups. Although we did not find any difference in 25( OH )D level between two groups in our study, high prevalence of vitamin D deficiency may influence these results and a clinical trial with vitamin D supplement therapy can be the next step of our study. 

## Conclusion

The first aim of the study was to find a relationship between vitamin D deficiency and PCOS, whereas the final result implicates to not only an association between vitamin D and metabolic syndrome, but also a real peril of pandemic of severe vitamin D deficiency which is considered as real threats for women of reproductive age. Although a direct association between PCOS and vitamin D was not found, it may need another study after the correction of vitamin D level. 
